# Microplastics-Assisted *Campylobacter* Persistence, Virulence, and Antimicrobial Resistance in the Food Chain: An Overview

**DOI:** 10.3390/foods14142432

**Published:** 2025-07-10

**Authors:** Irene Ortega-Sanz, Andreja Rajkovic

**Affiliations:** Department of Food Technology, Safety and Health, Faculty of Bioscience Engineering, Ghent University, Coupure Links 653, 9000 Ghent, Belgium; andreja.rajkovic@ugent.be

**Keywords:** biofilm, antibiotic resistance, horizontal gene transfer, environment, foodborne pathogen, food safety, campylobacteriosis

## Abstract

Recent studies have detected microplastics (MPs) in seafood and various food products worldwide, including poultry, fish, salt, beverages, fruits, and vegetables. This widespread contamination makes human exposure through consumption unavoidable and raises concerns for food safety and human health. MPs provide physical support to microorganisms for biofilm formation, protecting them from extreme conditions and facilitating their persistence in the environment. However, little is known about the impact of MPs in the transmission of foodborne pathogens and subsequent spread of infectious diseases like campylobacteriosis, the most common foodborne illness caused by a bacterium, *Campylobacter*. This review explores the sources of MP contamination in the food chain and offers a comprehensive overview of MP presence in animals, food products, and beverages. Moreover, we compile the available studies linking MPs and *Campylobacter* and examine the potential impact of these particles on the transmission of *Campylobacter* along the food chain with a particular focus on poultry, the main source and reservoir for the pathogen. While the environmental and toxicological effects of MPs are increasingly understood, their influence on the virulence of *Campylobacter* and the spread of antimicrobial resistance remains underexplored. Further studies are needed to develop standardized methods for isolating and identifying MPs, enabling comprehensive investigations and more effective monitoring and risk mitigation strategies.

## 1. Introduction

Microplastics (MPs) have emerged as a pervasive environmental contaminant that imperils our health and the planet. Found in diverse ecosystems, including marine and terrestrial environments, they are increasingly detected in food, water, and animal systems [1]. Simultaneously, *Campylobacter* species, the leading cause of bacterial gastroenteritis worldwide [2], thrives in similar environments. While other pathogens also pose risks, the unusual sensitivity of *Campylobacter* spp. to environmental stress suggests that additional factors, such as MPs, may contribute to the survival and persistence of the pathogen in the environment, potentially explaining its status as the most common cause of foodborne illness worldwide. This raises questions about possible interactions between MPs and bacterial pathogens, particularly their role in facilitating bacterial persistence, enhancing virulence, and exacerbating antimicrobial resistance (AMR) within the food chain.

Plastic pollution is a global environmental problem resulting from decades of unsustainable plastic production and improper disposal of plastic materials. From 1950 to 2023, global plastic production sharply increased from 2 to 413.8 million metric tons (Mt), with projections suggesting it will surpass 1.2 billion Mt by 2060 [3]. Once released into the environment, plastic waste undergoes degradation, resulting in smaller particles called MPs and nanoplastics (NPs). MPs are generally considered to be plastic particles ranging from 1 μm to 5 mm in size, while NPs are smaller than 1 μm [4]. These tiny particles have become a silent but significant global concern due to their ubiquity, long-term durability in the environment, and great potential for carrying and releasing toxic additives, harmful chemicals, and other pollutants [5,6]. Therefore, MPs and NPs represent a critical environmental challenge due to their increasing abundance and widespread distribution. This review will focus only on the role of MPs as transport vehicles for bacteria due to the inherent antibacterial properties of NPs [7].

While MPs’ environmental and toxicological impacts are becoming well documented [8,9,10], their interaction with foodborne pathogens remains largely unexplored. The irregular surface structure of MPs due to weathering forces (e.g., solar radiation, microbial action, and mechanical forces) serves as surfaces for bacterial attachment and colonization, driving the formation of biofilms [11]. In addition, biofilms protect bacteria against multiple extreme environmental factors, including biotic and abiotic factors like toxic substances, predation, and other environmental stress, such as temperature shifts, altered pH, osmotic stress, and antimicrobial or disinfectant agents [12]. Hence, bacteria become extremely resistant to cleaning and disinfection procedures in food production lines, compromising the effectiveness of cleaning strategies in biofilm removal. Therefore, MPs might pose a significant challenge to the food industry by serving as sources of foodborne pathogens with enhanced resistance capabilities, similar to their role in aquatic ecosystems [13,14].

The potential role of MPs in the transmission of foodborne pathogens may partly explain the global burden of foodborne diseases, which impacts more than 600 million people each year and results in major economic and social consequences [15]. Among these, *Campylobacter* infection (also called campylobacteriosis) is particularly significant, with over 148,000 human campylobacteriosis cases reported in the EU in 2023 [16]. However, the actual public incidence of the disease is believed to be 9 million per year in the EU due to non-reporting at a cost of around 2.4 billion euros to the public health systems [17]. Moreover, almost 90% of the human campylobacteriosis cases are caused by one species, *Campylobacter jejuni*, which remain a public health concern of global importance [18]. Poultry, particularly chickens, serves as the primary reservoir of *Campylobacter*, harbouring the bacteria in their gastrointestinal tract. During processing, defeathering and evisceration can introduce high bacterial loads into slaughterhouses and poultry processing plants, contaminating equipment and surfaces. Simultaneously, the use of plastic materials across the poultry supply chain serves as a source of MPs. Thus, the phenomenon of biofilm formation on these MPs may help explain how *Campylobacter* spp. are able to survive and persist throughout the entire food chain, from farm to fork, despite their high sensitivity to atmospheric oxygen concentrations [19], similar to what has been observed in aquatic ecosystems [20,21,22,23]. This could also account for the high prevalence of bacteria and the substantial number of reported campylobacteriosis cases. By providing a protective niche, biofilm formation on MPs may help this pathogen remain a notable global public health concern. This review critically examines the role of MPs as carriers of *Campylobacter*, exploring the mechanisms driving their interaction and their impact on bacterial survival and virulence. It also discusses the potential contribution of MPs to the proliferation of AMR in *Campylobacter*, with a particular focus on implications for food safety, aiming to provide a foundation for future research.

## 2. Microplastic Contamination in the Food Supply Chain

### 2.1. Sources of Microplastics

MPs are commonly classified into primary and secondary types based on their origin. Primary MPs refer to virgin plastic pellets intentionally manufactured for use in domestic and industrial products such as cosmetics, shower gels, and artificial turf [5]. Their small size allows them, among other things, to control the product’s viscosity, stability, and physical appearance and even provide an abrasive effect, acting as exfoliants. In contrast, secondary MPs result from the fragmentation of larger primary MPs or plastic items such as fishing nets, industrial resin pellets, household products, garments and home textiles, and discarded plastic debris, which gradually degrade over time due to exposure to weathering forces. UV-induced photo-oxidation, thermal degradation, hydrolysis, mechanical abrasion from waves and sand, and microbial activity are environmental factors influencing MP generation [24,25].

In food-related environments, there is evidence that food processing is a likely source of MP contamination [26]. Plastic materials found in water supply systems, feeding stations, transportation containers, conveyor belts, and worker clothes are potential sources of MP generation in the poultry supply chain. Furthermore, MPs can be released from plastic food packaging and contaminate food products, especially when the food containers are heated in microwave ovens [27,28,29,30]. Collectively, these scenarios underscore the growing concern over MPs as emerging contaminants and their potential to compromise food safety along the entire supply chain.

### 2.2. Distribution of Microplastics

Upon being released into the environment, MPs accumulate in the soil and on the shoreline, float across the waterways and through the air, sink in the water, and enter into the food chain through ingestion of contaminated feed, water, or surroundings by organisms [24,31,32,33,34]. The accumulation and persistence of MPs in the environment contribute to their toxicity, as their long lifespan allows them to remain in ecosystems for extended periods, leading to sustained exposure for organisms. When MPs enter the food chain, they can be transferred across trophic levels (e.g., marine food webs) and accumulate in animal organs, tissues, and cells through a process known as bioaccumulation, causing oxidative stress and cytotoxicity [10,35,36,37]. These effects have raised serious concerns about the widespread prevalence of MP across all environments, particularly for the food industry and human health, as these particles are increasingly making their way into the food chain and ultimately onto consumers’ plates [38]. While numerous studies have examined the presence of MPs in fish and fishery products, as well as in aquatic birds, there is a notable lack of research on terrestrial animals [38,39]. One of the few assessments includes the first evidence of MP transfer from soil to chickens reported in 2017 by Huerta Lwanga et al. [40]. To date, MPs have been found in various food products such as seafood, plant- and animal-based foods, beverages, and food additives [41,42]. In this context, this review provides an updated overview on MP contamination across animals, with a particular focus on poultry as the main reservoir of *Campylobacter*, as well as in food and beverages, underscoring the global scale of MP contamination (Table 1).

A great variation in MP content within the same type of sample is found across different locations and studies (Table 1), which might be attributed to differences in local plastic emissions [66,67]. Table sea salt, for instance, has been reported to contain between 16 and 681 MP particles/kg (Table 1), and drinking water, both tap and bottled, has shown high MP contamination rates globally, with 92% of samples testing positive in the USA and 72% in Europe [68]. Even more, tap water (TW) in the USA has the highest reported mean MP concentration of any country, with 9.24 ± 11.8 particles/L, while the lowest mean is observed in European Union (EU) countries at 1.68 ± 1.42 particles/L [61]. Higher levels of MP contamination are frequently reported in bottled water (BW) than in TW, particularly in reusable polyethylene terephthalate (PET) bottles [69]. Nevertheless, difficulties in separating MPs may also account for these variations, as current analytical methods can partially or completely degrade the particles [70], and standardized isolation protocols are lacking, highlighting the urgent need for harmonized analytical approaches [69].

MPs can be transferred to poultry products during processing through various mechanisms, including the use of MP-contaminated table sea salt and water, which are used for seasoning or washing raw chicken, as commonly practiced in various cultures before cooking. This raises important concerns in the context of food safety as food additives and water represent a significant route for MP transfer to poultry meat. This is especially relevant considering that both water and cooking salt have been shown to carry *Campylobacter* spp. [71,72]. Thus, the presence of MPs in these samples may act not only as physical contaminants but also as potential carriers for *Campylobacter* during domestic food handling, increasing the risk of cross-contamination and persistence on surfaces. Moreover, contact with plastic equipment and the breakdown of plastic packaging can result in the transfer of MPs to poultry meat [26]. For example, polystyrene (PS) tray packaging-derived MPs (300–450 μm) were observed in packaged poultry products, including chicken breast and turkey escalope [73]. Besides, studies have reported the cross-contamination of chicken breast meat from plastic cutting boards (8.24–1454.5 μm) [74]. This provides evidence that MPs can also reach food products upon release from plastic surfaces or food packaging during production, storage, and transportation [28,75], albeit depending on the type of packaging and brand, thereby raising concerns about the food safety of plastic packaging. These findings underscore the growing threat that MPs pose to food safety and emphasize the potential risks associated with human exposure through the consumption of contaminated food.

## 3. Interaction Between Microplastics and *Campylobacter*

### 3.1. Biofilm Formation and Persistence

**Figure 1:**
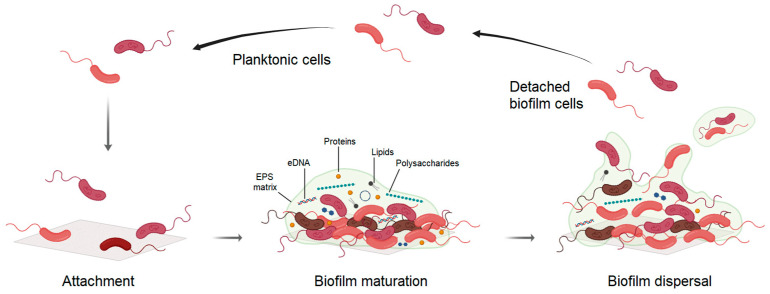
*Campylobacter* biofilm formation on microplastic (MP) surfaces. Schematic illustration of the three main stages of biofilm formation: initial bacterial adhesion, biofilm maturation, and dispersion. Free-floating (i.e., planktonic) bacteria can encounter an MP surface and attach within minutes. Once attached, they begin to produce slimy extracellular polymeric substances (EPS), initiating surface colonization. EPS production facilitates the development of a complex, three-dimensional biofilm structure within hours that comprises polysaccharides, secreted proteins, lipids, water, and extracellular DNAs, such as plasmids. Biofilms can propagate through the detachment of cell clumps or individual cells, which can then colonize new MP surfaces.

MPs existing in nature are subjected to weathering forces that modify their surface structure [11]. Environmental factors like mechanical forces, solar radiation, and chemical and thermal oxidation increase the specific surface area of MPs and their surface hydrophobicity, thereby enhancing their susceptibility to microbial colonization and absorption of other contaminants like heavy metals, toxic additives, or organic pollutants [5]. Such modified surface characteristics of MPs provide a unique substratum for microbial attachment, including spoilage bacteria and foodborne pathogens like *Campylobacter* spp., ultimately resulting in biofilm formation (Figure 1). Biofilms are complex immobile communities of microbes encased in self-produced matrix of polysaccharides, secreted proteins, and extracellular DNAs (eDNAs), which offer protection to the bacteria from environmental stressors (temperature, disinfectants, microbial competition, etc.) [76]. As a result, bacterial survival is enhanced, and environmental persistence is prolonged. Several studies have reported increased tolerance of *C. jejuni* cells encased in a biofilm matrix to stresses than their planktonic counterparts, prolonging the survival up to 24 days longer under aerobic conditions [77,78,79]. Therefore, given the sensitivity of *Campylobacter* to oxygen and their enhanced ability to form biofilm under aerobic conditions on plastic surfaces compared to other materials such as stainless steel and copper [19,77,79], MPs may serve as favourable substrates that promote *Campylobacter* survival under natural environmental conditions. In fact, studies show that MPs exhibited a higher propensity for facilitating biofilm than other materials such as glass [80]. Likewise, bacteria can detach from the biofilm and disperse, colonizing new niches [81]. In this context, MPs act not only as surfaces for biofilm formation but also as vehicles that facilitate the environmental transport of *Campylobacter*. By moving through water currents or being carried by wind or animal activity, MPs can disseminate the attached pathogen across both aquatic and terrestrial ecosystems. This mobility contributes to the environmental persistence of *Campylobacter*, potentially introducing it into new habitats where it might not otherwise exist. Moreover, MPs select bacterial cells that are better at forming biofilms [80]. Consequently, the formation of biofilms by *C. jejuni* on MPs not only enhances its persistence in various environments but also poses a significant risk for contamination and transmission, particularly in food processing settings.

#### Studies on *Campylobacter* Associated with Microplastics

Despite the high risk of *Campylobacter*-associated biofilm formation on MPs in the poultry supply chain, the relevance of MPs in the transmission route of *Campylobacter* to cause infection and disease in humans remains largely unexplored. This gap of knowledge arises from (1) the notorious difficulties to isolate, grow, and identify the bacteria and (2) lack of standard methods in MP research. Evidence of *Campylobacter* colonization on MPs was first reported in 2014, when it was detected in wastewater effluents [20] (Table 2). More recently, *Campylobacter* has also been identified on MPs recovered from seawater in the northern Adriatic Sea [22]. Furthermore, *Campylobacter* has also been identified in sand samples collected from beaches in Scotland (401 ± 46 CFU/100 g dry weight sand), where MP particles were also found, suggesting that MPs may have played a role in transporting the pathogen to these environments [82]. The latest evidence comes from a 21-day field incubation study conducted in coastal waters of Hong Kong (China) that demonstrated the ability of Campylobacteria to colonize polypropylene (PP), polyethylene (PE), and PS particles [23]. However, their abundance varied depending on the water site and polymer type (PP, 3.9%; PP + PE, 4.8%; PS, 3.0%) (Table 2). Notably, a much higher abundance of the pathogen was observed during the early stages of biofilm formation (day 3), reaching 20.4%, 25.4%, and 5.9% on PP, PP + PE, and PS particles, respectively. The authors suggested that Campylobacteria may exploit early ecological niches, rapidly colonizing them before decreasing in abundance as other bacterial groups become established within the biofilm. So far, the occurrence of *Campylobacter* on MPs has only been reported in aquatic environments, where the bacterium is considered an emerging concern due to increasing anthropogenic activities [83]. However, the specific abundance of *Campylobacter* cells per MP particle in natural environments has not yet been determined. The only study to date quantifying bacterial concentration on MP surfaces has been recently conducted in vitro by Ortega-Sanz et al. [84], demonstrating that *C. jejuni* NCTC 11,168 can reach levels between 5.4 and 6.5 log CFU/cm^2^ after 24 h and 72 h of biofilm formation, respectively. Hence, there is an urgent task of understanding how MPs interact with this infectious disease agent to further measure the impact of MPs on the survival and persistence of the pathogen in the environment and along the poultry supply chain in particular, where the bacteria are highly persistent [85]. Research focused on the ability of *Campylobacter* to form biofilm on different types of MP particles is also recommended, considering the influence of polymer type on biofilm formation [23,79].

### 3.2. Virulence

Weathering of MPs results in the formation of cracks on the MP surface that facilitate the accumulation of environmental pollutants, such as toxic additives, heavy metals, and organic contaminants, including antibiotics [86,87,88]. These MP-associated pollutants induce oxidative stress on bacteria, triggering stress responses that promote biofilm formation on MPs to provide protective environments [89,90]. Exposure to increased iron concentrations commonly results in the accumulation of total reactive oxygen species (ROS) in *C. jejuni*, which increases the production of eDNA and polysaccharides for the production of extracellular polymeric substance (EPS) and biofilm formation [91]. Similarly, presence of specific antibiotics (ampicillin, ciprofloxacin, erythromycin, nalidixic acid, rifampicin, and tetracycline) induces biofilm formation in sensitive *C. jejuni* strains [92]. Hence, the colonization of MPs with adsorbed contaminants by *Campylobacter* may thrive the persistence of the pathogen in the environment with improved fitness, particularly those strains with metal resistance or antibiotic resistance genes. In addition, favourable environmental conditions or adverse conditions inside the biofilm can trigger biofilm dispersion (Figure 1), such as the concentration of signal molecules or increased oxygen levels, which results in the dissemination of biofilm cells with improved abilities to survive in diverse environments [93].

**Figure 2:**
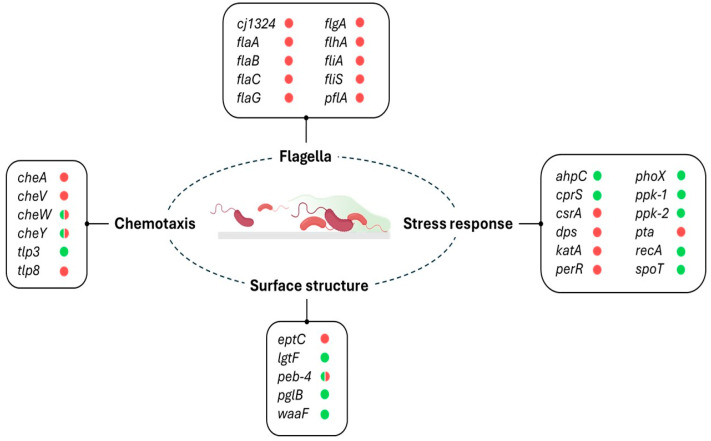
Potential *Campylobacter* genes and pathways involved in biofilm formation on MPs. The coloured circles indicate whether a mutant version of the gene increases (green) or reduces (red) biofilm formation on larger surfaces. Circles containing both colours indicate that different studies have reported opposing effects for the same gene [95].

Approximately 600 *Campylobacter* genes are differentially expressed during biofilm formation, involving pathways related to motility (flagellins *flaA* and *flaB*), iron metabolism (enterochelin uptake, *ceuBCD*; and haemin uptake, *chuABCD*; *cfrA*), cell division and peptidoglycan (*pbpAC*, *murG*, and *mreB*), and synthesis of lipooligosaccharide (*waaF* and *lgtF*) and N-glycans (*pglABCDIG*), among others [94]. However, it remains unclear which of these genes actively contribute to biofilm formation and which are instead affected as a result of the altered physiological state within mature biofilms [95]. This uncertainty is further compounded by the high variability in the gene repertoire among *Campylobacter* strains capable of forming biofilms [96]. Nevertheless, several genes are known to influence the biofilm-forming capacity of *C. jejuni*, including *luxS*, which regulates biofilm formation through the synthesis of the quorum-sensing molecule autoinducer-2 (AI-2) [95]. A summary of these genes is presented in Figure 2. These genes may serve as potential targets for controlling biofilm development on MPs, although further research is needed to clarify whether these genes or pathways are specifically involved in biofilm formation on these surfaces. Nonetheless, there is no reason to assume that the molecular mechanisms governing biofilm formation on MPs differ substantially from those on other abiotic surfaces. Therefore, it is likely that the same genetic pathways play a key role in enabling *Campylobacter* to adhere to and colonize MPs, facilitating their environmental persistence and dissemination.

#### Impact of Microplastic-Associated Pollutants on *Campylobacter* Virulence Gene Expression

As a microaerophilic bacterium, *Campylobacter* has unique mechanisms to face oxidative stress and remove ROS. Opposite to other bacteria like *Escherichia coli* and *Salmonella*, the pathogen possesses only a sole copy of genes encoding ROS detoxification enzymes, such as alkyl hydroperoxide reductase (*ahpC*) and catalase A (*katA*) for the detoxification of H_2_O_2_, and superoxide dismutase (*sodB*) for the detoxification of superoxide [97]. In addition, the expression of these genes is mainly modulated through the coordinated transcription of the regulators PerR (peroxide response regulator) and CosR (*Campylobacter* oxidative stress regulator) [98] instead of the typical oxidative defence systems of many bacterial species, namely the SoxRS, OxyR, and RpoS regulons [99]. However, the exact changes in *Campylobacter* gene expression that promote the initial attachment of cells under stress for biofilm formation, followed by biofilm maturation and dispersion, are poorly understood. Nonetheless, the oxidative stress response induces the expression of specific virulence genes as part of bacterial survival strategies [100].

The *Campylobacter* CadF (*Campylobacter* adhesin to fibronectin) and FlpA (fibronectin-like protein A) fibronectin-binding proteins and JlpA (Jejuni lipoprotein A) play a central role in biofilm formation, serving as critical factors for initial cell adhesion and invasion [101]. These adhesins are essential virulence factors for the pathogenesis of *Campylobacter* to adhere, colonize, and invade host cells. The *cadF* gene was typically upregulated in *C. jejuni* (80% of strains) under exposure to hydrogen peroxide, although the change in gene expression was not always correlated with increased adhesion or invasion [100]. Similarly, chitosan at subinhibitory concentrations (0.0125%) upregulated specific genes for stress response (*sodB*) and attachment (*ciaB, jlpA*), increasing the adhesion of the bacteria to surfaces and potential for biofilm formation under stress conditions [102]. CiaB (*Campylobacter* invasion antigen B) is a secretion protein that contributes to host–cell interactions and pathogen survival within the host [103]. Likewise, the presence of carvacrol at the subinhibitory level (0.002%) significantly upregulated the *cetB* gene, which mediates energy taxis responses essential for motility in response to stimuli, attachment, and biofilm formation on various surfaces [104,105,106]. Unfortunately, no studies to date have examined the impact of MPs on the expression of *Campylobacter* virulence genes, limiting our understanding of the potential role that MPs may play in modulating the pathogenicity of this pathogen. Nonetheless, the lack of data does not imply that MPs fail to contribute to *Campylobacter* virulence. In fact, previous findings demonstrate that biofilm formation on larger surfaces increases the expression of *Campylobacter* virulence genes, underscoring the urgent need for further research with MPs. This gap in knowledge likely results from the scarcity of specialized methods for isolating high-quality RNA from MPs typically due to limited bacterial cell quantities and interference from MP particles during downstream purification. Once these technical challenges are addressed, genes such as *cadF*, *sodB*, *ciaB*, *jlpA*, and *cetB*, among others, represent suitable targets for directly investigating how the presence of MPs affects *Campylobacter* gene expression and, consequently, the virulence of the pathogen.

Quorum sensing has also been proposed to be responsible for upregulating genes associated with the oxidative stress response. LuxS-deletion mutants expressed significantly lower levels of *ahpC* and *tpx* and displayed significantly reduced biofilm formation compared to the wild-type *C. jejuni* 81–176 strain [107,108]. This suggests that *luxS*, encoding an AI-2 biosynthesis enzyme, could also aid biofilm formation on MPs by *C. jejuni* under stress conditions. Quorum sensing facilitates biofilm formation by regulating genes associated with flagellation and motility. In *Campylobacter luxS* mutants, the bacteria exhibit reduced transcription of the *flaA* gene [109], which encodes one of the two flagellins. Thus, flagellation and flagellin glycosylation might also be essential processes for biofilm formation on MPs [105].

While upregulation of virulence genes has been demonstrated in some studies, varying expression patterns can be observed in the cytolethal distending toxin genes, *cdtA*, *cdtB*, and *cdtC*, between strains [100]. The *cdtABC* operon is responsible for the expression of a cytolethal distending toxin (CDT), which disrupts mucosal barriers by inducing host–cell apoptosis [110]. In the study by Koolman et al. [100], only *cdtB* and *cdtC* showed a significantly different (*p* < 0.05) transcriptomic response in *C. jejuni*. The *cdtB* gene was significantly downregulated in 30% of the strains, including the reference *C. jejuni* NCTC 11,168 strain, while significantly upregulated in 1 out of 10 strains. Conversely, the *cdtC* gene was significantly downregulated in another strain. Both *cdtA* and *cdtC* are critical for toxin binding to the host cell, whereas CdtB is translocated into the host cell membrane, where it induces cell cycle arrest at the G2/M phase, ultimately leading to cell death [111]. These gene expression studies suggest that the virulence gene expression changes occurring in *Campylobacter* during biofilm formation on MPs might be strain-dependent, with no direct promotion of the virulence of the pathogen or their ability to invade host cells. However, further studies are recommended to confirm this hypothesis, such as cell invasion assays using Caco-2 cells, especially considering that *Listeria monocytogenes* biofilm cells adhered to MPs exhibit increased virulence compared to both free-living cells and those attached to glass surfaces [112]. These findings demonstrate the impact of biofilm formation on *Campylobacter* virulence gene expression, as well as the role of MPs in modulating foodborne pathogens, providing a valuable basis for identifying potential virulence genes that could be further investigated in *Campylobacter* to elucidate how MPs influence the virulence of the pathogen.

### 3.3. Influence of Microplastics on Campylobacter Antimicrobial Resistance

In many countries, antibiotics are overused in poultry farming, accounting for an estimated 73% of total antibiotic consumption [113,114]. This excessive use has significantly contributed to the growing problem of antibiotic resistance and has also led to the presence of antibiotics residues in animal feed and environment, compromising both human and animal health [115]. Like other pollutants, antibiotics can adsorb onto MPs, creating hotspots for not only resistance selection but also for spread of resistant genes [116]. Moreover, antibiotics can accumulate on MPs at levels exceeding the environmental background [117]. Ultimately, these MP-associated antibiotics can be transferred to organisms and even humans through the food chain, compromising antimicrobial therapy. Antibiotics such as amoxicillin (β-lactam), ciprofloxacin (fluoroquinolone), and tetracycline have been reported to adsorb onto MPs, although at adsorption levels varying according to the type of plastic and particle size [117,118,119]. To date, the actual impact of antibiotics adsorbed onto MPs on the spread of *Campylobacter* resistance remains unexplored, although this phenomenon may contribute to the elevated incidence of β-lactam-, fluoroquinolone-, and tetracycline-resistant *Campylobacter* strains in food-producing animals [120]. Alternatively, the antibiotics can desorb from the surface of the MPs, resulting in the dissemination of the antibiotics in the environment. Similarly, the desorption rate was demonstrated to vary depending on the type of plastic, with PE favouring the desorption compared to PS and polyvinyl chloride (PVC) [121].

The biofilm matrix can also impede antimicrobial penetration, leading to increased resistance. Multiple studies have demonstrated that significantly higher concentrations of antimicrobials are necessary to eradicate *Campylobacter* biofilms than to eliminate planktonic cells [122,123]. For example, resistance to gentamicin in biofilm-associated *C. jejuni* was found to be up to 32 times higher than in corresponding planktonic cells [124]. Whether MPs can also replicate this effect remains unknown, as no study has yet evaluated how the MP particles influence antibiotic resistance in this pathogen. Furthermore, exposure of *Campylobacter* biofilm cells to sublethal antibiotic levels results in constant selective pressure that triggers adaptive responses in the bacteria, including the emergence of spontaneous mutations conferring antibiotic resistance, gene recombination, and horizontal gene transfer (HGT) [125]. These antibiotic resistance mechanisms developed by *Campylobacter* are summarised in Table 3. Natural transformation, conjugation (i.e., cell-to-cell contact), and transduction (i.e., bacteriophages) are key HGT mechanisms that enable *Campylobacter* to acquire resistance genes from other bacteria, thereby accelerating the dissemination of AMR [126]. For example, under laboratory conditions, engineered *C. jejuni* cells were able to transfer antibiotic resistance genes such as *aphA-3* and *cat*, conferring resistance to kanamycin and chloramphenicol, respectively, to other *C. jejuni* biofilm-associated cells through natural transformation, which were subsequently released into the environment [127,128]. This confirms that HGT within *Campylobacter* biofilms contributes to AMR evolution. Other antibiotic resistance gene known to be transferred through HGT is *tet* (O), which is particularly notable for conferring tetracycline resistance through plasmid-mediated conjugation [129]. However, it remains unclear whether these mechanisms contribute to the development of AMR in *Campylobacter* cells adhered to MPs. Therefore, there is an urgent need to first investigate the extent to which the presence of MPs influences antibiotic resistance in *Campylobacter*, particularly in light of evidence showing that MPs promote multidrug resistance in foodborne pathogens. Gross et al. [80] reported that *E. coli* cells within MP biofilms had elevated multidrug resistance within 5 to 10 days of exposure to four antibiotics (ampicillin, ciprofloxacin, doxycycline, and streptomycin), regardless of their size or concentration. Therefore, a similar effect may arise from the colonization of MPs by *Campylobacter* when exposed to antibiotics. To evaluate biofilm-specific resistance in *Campylobacter* biofilms adhered to MPs, careful consideration must be given to the methodology used. Sonication, the most efficient method to detach culturable bacterial biofilms [130], generates heat that may release nucleic acids and antibiotic resistance genes [131], potentially leading to misleading results.

In addition, the closer contact among bacterial cells within the biofilm matrix promotes the exchange of eDNA, conjugative plasmids, and conjugative transposons between donor and receptor cells compared to free-living, planktonic bacteria. While several bacterial genera (*Staphylococcus*, *Enterococcus*, *Streptococcus*, *Pseudomonas*, and *Salmonella*) have shown gene transfer rates up to 1000 times higher in biofilms compared to their planktonic state, *C. jejuni* exhibits significantly lower increases in gene transfer within biofilms compared to planktonic cells (up to 17.5-fold) [127,132,133,134]. Moreover, Svensson et al. [135] reported the emergence of dual-resistant *C. jejuni* in biofilms exposed to sublethal concentrations of bile salts, inducing oxidative damage, which triggered a higher release of eDNA in the biofilm matrix compared to single-resistant strains. The eDNA released in the environment that favours biofilm development also contributes to the spread of antibiotic resistance in *Campylobacter* [128], highlighting its potential to serve as a substrate for HGT on MPs. Therefore, the interaction between *Campylobacter* biofilms and MPs represents a potential hotspot for the spread of AMR. Understanding the extent and nature of HGT in *C. jejuni* biofilms formed on MPs is crucial for assessing their role in AMR dissemination and developing effective mitigation strategies.

**Table 3:** *Campylobacter* antibiotic resistance mechanisms potentially driven by MPs.

Antibiotic Class	Resistance Mechanism	Reference
Aminoglycosides (gentamicin, amikacin, kanamycin, netilmicin, spectinomycin)	*aac(3)* *aac(6′)-Ib* *aph(2″)-Ig* *aph(2″)-If* *aphA-3* *sat-4* *ant6* *ant2* *antA* *antB* ***rpsL***	[136]
β-lactams (penicillin)	Enzymatic inactivation of the antibiotic by ***blaOXA*** genes Efflux through multidrug efflux pumps like C*meABC*	[137,138,139]
Chloramphenicol (chloramphenicol)	***cat***	[140]
Fluoroquinolones (ciprofloxacin)	Point mutations in GyrA (T86I, T86K, A70T, D90N, **P104S**) Efflux through multidrug efflux pump CmeABC	[138,141,142]
Lincosamides (clindamycin)	*lnu(AN2)* *lnu(C)*	[139,143]
Macrolides (erythromycin)	Point mutations in 23S rRNA (A2074C, A2074G, A2075G) and/or ribosomal proteins L4 (V196A, S2R, V121A, I200F, M192I) and L22 (I65V, S109A, A103V, A74G, S109T, E111A, T114A, K15I) ***erm*****(B)** Efflux through multidrug efflux pump CmeABC	[138,144,145,146]
Tetracyclines (tetracycline)	***tet*****(O)**-like genes Efflux through multidrug efflux pump CmeABC	[138]

Genes in bold indicate evidence of HGT [127,144,147].

### 3.4. Co-Aggregation of Campylobacter with Other Bacterial Species

In natural environments, biofilms are most commonly composed of multispecies microorganisms (i.e., mixed-species biofilms). The presence of other bacterial species in the environment, such as *E. coli*, *Pseudomonas aeruginosa, Enterococcus faecalis*, or *Staphylococcus simulans*, facilitates the formation of biofilm on plastic by *C. jejuni* [148,149,150]. By creating localized microaerophilic conditions within the biofilm, these bacteria originating from poultry sources provide favourable conditions for the survival of *C. jejuni* in food-related environments, ultimately promoting their persistence in the environment. Since *Campylobacter* species exhibit limited survival outside the host, it is unlikely that they act as the initial colonizer in biofilm formation on MPs. In fact, *C. jejuni* is a poor biofilm initiator and mainly functions as a secondary colonizer of biofilms on plastic previously established by other species [149,151]. Therefore, it is reasonable to hypothesize that the co-aggregation of *Campylobacter* with other bacterial species on MPs could contribute to its persistence in the poultry supply chain, facilitated by the presence of other supporting bacterial species. Notably, colonization of MPs by foodborne pathogens such as *E. coli* and *P. aeruginosa* has been well documented [152,153], including evidence of direct association between *P. aeruginosa* and *Campylobacter* on MPs in marine environments [22]. This suggests that mixed-species biofilms on MPs involving *Campylobacter* could also develop in poultry processing environments. Moreover, the high prevalence of early colonizers such as *E. coli*, *E. faecalis*, and *P. aeruginosa* on MPs, persisting for at least 25 days and accumulating in greater numbers than on glass particles, potentially facilitates the biofilm formation of *Campylobacter* on these particles and enhances their persistence within the poultry supply chain [154].

## 4. Implications for the Food Chain

The widespread presence of MPs poses a significant threat to humans and food safety. As MPs are generated within food-related environments, their coexistence with *Campylobacter* raises serious concerns. *Campylobacter* cells that attach to MPs may evade standard decontamination procedures in food processing facilities, potentially persisting on raw food surfaces and in water supplies. This issue is further compounded during the storage of *C. jejuni*-contaminated poultry meat at refrigeration temperatures, where the bacteria can develop cold tolerance [155]. Such adaptations not only enhance their survival but also result in strains with increased virulence that are more likely to cause human infections. The presence of MPs in packaged chicken meat may offer protective niches that support bacterial survival under refrigeration, thereby facilitating *Campylobacter* persistence along the food chain. Therefore, the combination of MP contamination in the food chain and the adaptive capability of *Campylobacter* underscores the urgent need for improved food safety strategies.

To date, the presence of *Campylobacter* attached to MPs has been documented exclusively in aquatic environments, indicating a potential route for bacterial dissemination through water systems (Table 2). This elevates the risk of both seafood contamination and the transfer of *Campylobacter* to plant-based foods through irrigation water polluted with MPs carrying the pathogen. This consequently heightens human exposure to *Campylobacter*. Additionally, cross-contamination events during poultry processing or food handling can increase the risk of campylobacteriosis. Scientific evidence indicates that plastic cutting boards are significant potential vehicles for the cross-contamination of the pathogen from raw chicken to ready-to-eat foods [156]. Additionally, these boards can release MPs, which may serve as surfaces for biofilm formation, resulting in biofilm-associated MPs that can subsequently spread throughout kitchen environments, further increasing the risk of *Campylobacter* infection in humans. A similar process may occur with plastic components of agricultural and industrial equipment. These findings highlight the potential role of MPs as carriers of *Campylobacter* through the food chain, raising concerns about their impact on food safety and public health.

## 5. Future Directions

Standardization of procedures for the isolation, identification, and quantification of MPs in poultry meat products should be developed to guarantee uniformity across different studies. This requires interdisciplinary collaboration, involving analytical chemistry, environmental science, food science, and microscopy expertise. Besides, future studies should aim to identify the specific origins of MPs throughout the poultry supply chain, from farming and production to processing, packaging, and domestic environments. Nonetheless, studies on the environmental fate of MPs are also crucial to understand how MPs enter the food chain. This knowledge can help target MP-related risks in food systems, enabling more effective intervention strategies to reduce MP contamination, which in turn may lower the risk of *Campylobacter* transmission. However, these efforts should also be supported by public awareness campaigns aimed at actively educating manufacturing companies and local communities on the responsible use of plastics. These initiatives can promote global action on reuse, recycling, and reduction of plastic while also influencing consumer choices, potentially leading to decreased MP generation.

Moreover, mechanistic studies should focus on elucidating how MPs influence *Campylobacter* physiology, including biofilm dynamics and gene expression. Understanding the relationship between changes in biofilm biomass over time and metabolic activity is key to develop efficient biofilm control strategies. Investigating how MPs modulate the expression of the genes associated with biofilm formation could reveal novel pathways by which these pollutants contribute to enhanced *Campylobacter* persistence and pathogenicity, ultimately helping to target mitigation approaches. In addition, studies are needed to assess the role of MPs in the spread of AMR in order to support the development of effective strategies to combat MP-associated AMR. Collectively, this emerging research will provide valuable insights into the contribution of MP-associated *Campylobacter* to foodborne illnesses, which will contribute significantly to the advancement of quantitative risk assessments.

## 6. Conclusions

MPs represent an emerging challenge in food safety, particularly as they intersect *Campylobacter* survival, virulence, and AMR. Despite the widespread environmental presence of both MPs and *Campylobacter*, their interaction remains poorly understood. Only a limited number of studies have reported their co-occurrence in the environment, underscoring the potential of MPs to act as vehicles for *Campylobacter* contamination and subsequent human infection. This highlights the urgent need for in-depth characterization of the interaction between MPs and *Campylobacter*, particularly in poultry-related environments, with a focus on how these interactions may enhance pathogen virulence and facilitate the spread of AMR. Addressing this complex threat requires an integrated approach that includes environmental monitoring, advanced food processing technologies, and enhanced consumer awareness. While significant progress has been made in addressing the MP issue in recent years, the development of standard experimental techniques for their separation and identification will enhance our ability to study their role in *Campylobacter* contamination. This will further enable comprehensive investigations and the development of effective risk mitigation strategies.

## Figures and Tables

**Table 1:** Microplastic (MP) levels in animals, food, and beverages. Details include the sample type analysed, the country where MPs were found, abundance of MPs found, analytical method used to measure MPs, types of MPs, identified, and reference to the respective study.

Sample	Country	Extraction Method	Quantification Method	MP Concentration	Identification Method ^1^	MP Type ^2^	Reference
Poultry	Mexico (home garden)	After flotation in demineralized water	Stereo microscope	129.8 ± 82.3 particles/g chicken faeces	Not determined	NA	[40]
	Mexico (home garden)	After flotation in demineralized water	Stereo microscope	10.2 ± 13.8 particles/g chicken gizzards	Not determined	NA	[40]
	China	Lyophilization, followed by digestion with 30% H_2_O_2_ and iron catalyst solution prepared with 20 g iron (II) sulfate heptahydrate in 1 L ultrapure water	Not determined	Identified in chicken faeces	Raman	PA and PET	[43]
	Indonesia	Digestion solution (10 N KOH) [44]	Stereo microscope	27 to 49 particles/duck	Unknown	PE, PET, PVC, PBM, and PA	[45]
Commercial fish	Malaysia	Digestion solution (10% KOH) [44]	Stereo microscope	0 to 10 particles/individual	µ-Raman	PE	[46]
	Portugal	Visual inspection, followed by digestion solution (10% KOH)	Stereo microscope	1.67 ± 0.27 particles/individual	µ-FTIR	PES and PP	[47]
Commercial dried fish	Sri Lanka	Digestion solution (10% KOH)	Stereo microscope	0.96 ± 0.17 particles/individual	µ-FTIR	PE, PS, and PVC	[48]
Mussels	Vietnam (green mussels)	Digestion solution (10% KOH) and saturated NaI solution	Stereo microscope	3.3 ± 2.4 particles/individual	ATR-FTIR	PA, PAA, PET, PS, PE, and PP	[49]
	Northern Tunisia (Mediterranean mussels)	Digestion solution (10% KOH) [50]	Stereo microscope	2.6 ± 1.7 to 12.0 ± 1.4 particles/individual	ATR-FTIR	PE and PP	[51]
	UK (blue mussel)	Digestion solution (30% H_2_O_2_) [52]	Stereo microscope	1.1 to 6.4 particles/individual	µ-FTIR	PET and PES	[53]
Oysters (*Crassostrea gigas*)	Vietnam	Digestion solution (10% KOH and 30% H_2_O_2_) [50,54]	Unknown	18.54 ± 10.08 particles/individual	µ-FTIR	PA, EVOH, PF, PTFE, and PEI	[55]
	USA	Digestion solution (30% H_2_O_2_) [56]	Stereo microscope	0.69 to 3 particles/individual	µ-FTIR	PS, PE, PP, PC, and polyacrylate	[57]
	Spain	Digestion solution (2 M KOH + 10% SDS), followed by enzymatic hydrolysis (protease, lipases, and celluloses), oxidation with 33–35% H_2_O_2_, peroxide oxidation (Fenton processes), and enzymatic hydrolysis with chitinase	Stereo microscope	22.8 ± 14.4 particles/individual	ATR-FTIR and μFTIR	PE and PES	[58]
Carrots	Italy	Blended, dried, followed by digestion (65% nitric acid)	SEM	101,950 ± 44,368 particles/g	Not determined	NA	[59]
Lettuce	Italy	Blended, dried, followed by digestion (65% nitric acid)	SEM	50,550 ± 25,011 particles/g	Not determined	NA	[59]
Broccoli	Italy	Blended, dried, followed by digestion (65% nitric acid)	SEM	126,150 ± 80,715 particles/g	Not determined	NA	[59]
Apple	Italy	Blended, dried, followed by digestion (65% nitric acid)	SEM	195,500 ± 128,687 particles/g	Not determined	NA	[59]
Pear	Italy	Blended, dried, followed by digestion (65% nitric acid)	SEM	189,550 ± 105,558 particles/g	Not determined	NA	[59]
Industrial beer	Ecuador	Digestion solution (30% H_2_O_2_)	Inverted microscope (10x)	47 particles/L	FTIR	PP, PE, and PAM	[60]
	USA	11 µm membrane filtration	Stereo microscope	4.05 particles/L (0 to 14.3 particles/L)	Not determined	NA	[61]
Bottled water	USA	2.5 µm membrane filtration	Stereo microscope	3.57 ± 1.79 particles/L (1.78 to 5.37 particles/L)	Not determined	NA	[61]
Bottled water (PET)	Mexico	1.5 µm glass fiber filtration	Fluorescence microscope	686 particles/L (11 to 2267 particles/L)	FTIR	PP, PA, PS, PE, and PEST	[62]
	India	1.5 µm glass fiber filtration	Fluorescence microscope	213 particles/L (2 to 1810 particles/L)	FTIR	PP, PA, PS, PE, and PEST	[62]
Tap water	USA	2.5 µm membrane filtration	Stereo microscope	9.24 ± 11.8 particles/L (0 to 60.9 particles/L)	Not determined	NA	[61]
	Ecuador	2.5 µm membrane filtration	Stereo microscope	4.02 ± 3.20 particles/L (0 to 9.04 particles/L)	Not determined	NA	[61]
	Germany	2.5 µm membrane filtration	Stereo microscope	0.91 ± 1.29 particles/L (0 to 1.82 particles/L)	Not determined	NA	[61]
	Worldwide	2.5 µm membrane filtration	Stereo microscope	5.45 particles/L (0 to 60.9 particles/L)	Not determined	NA	[61]
Table sea salt	China	Digestion solution (30% H_2_O_2_)	Stereo microscope	550 to 681 particles/kg	µ-FTIR	PET, PE, PES, PB, PP, and PAN	[63]
	Spain	5 µm membrane filtration	Stereo microscope	50–280 particles/kg	FTIR	PET, PP, and PE	[64]
	Turkey	Digestion solution (30% H_2_O_2_), followed by 0.2 µm membrane filtration, and digestion with 4 M NaI solution	Stereo microscope	16–84 particles/kg	µ-Raman	PE and PP	[65]

^1^ ATR-FTIR, attenuated total reflection-fourier transform infrared spectroscopy. ^2^ EVOH, ethylene-vinyl alcohol; PA, polyamide (nylon); PAA, polyacrylic acid; PAM, polyacrylamide; PAN, polyacrylonitrile; PB, polybutylene; PBM, poly-(n-butyl methacrylate); PC, polycarbonate; PE, polyethylene; PEI, polyetherimide; PES, polyethersulfone; PEST, polyester + polyethylene terephthalate; PET, polyethylene terephthalate; PP, polypropylene; PS, polystyrene; PTFE, polytetrafluoroethylene; PVC, polyvinyl chloride; NA, not applicable.

**Table 2:** Reported cases of *Campylobacter* detected on MPs worldwide.

Country	Sample Type	MP Detection Method	MP Quantification Method	Bacterial Detection Method	Class/Family/Genus/Strain	Abundance	Reference
USA	Wastewater effluents	0.33–2 mm membrane filtration, followed by peroxide oxidation (0.05 M Fe (II) and 30% hydrogen peroxide) and a salinity-based density separation	Stereo microscope	16S rRNA gene sequencing	*Campylobacteraceae*	7.4%	[20]
USA	Wastewater treatment plants (WWTPs)	0.3 mm membrane filtration, followed by peroxide oxidation (0.05 M Fe (II) and 30% hydrogen peroxide) and a salinity-based density separation	Stereo microscope	16S rRNA gene sequencing	*Campylobacteraceae* (94% assigned to *Arcobacter*)	~11% sewage <1% effluent ~1% sludge	[21]
Slovenia	Seawater in the northern Adriatic Sea	Digestion solution (10% KOH), followed by 20 μm membrane filtration	Stereo microscope	16S rRNA gene sequencing	*Campylobacter*	>30%	[22]
China	Seawater in four coastal sites of Hong Kong, namely the Ma Wan fish farm (FF), Ma Wan Beach (Beach), the Hong Kong University of Science and Technology Pier (Pier), and Yau Ma Tei typhoon shelter (TS) after 21 days incubation	Manually collected from nylon pouches where they had been deliberately placed	NA (10 MP pellets per nylon pouch)	16S rRNA gene sequencing	Campylobacteria	FF: < 0.5% Beach: < 0.5% Pier: < 0.5% TS: 3.9%	[23]
Belgium	5 mm PET discs (in vitro)	Self-prepared	NA	Colony counting	*C. jejuni* NCTC 11168	5.4 to 6.5 log CFU/cm^2^	[84] (in preparation)

NA, not applicable.

## Data Availability

No new data were created or analyzed in this study. Data sharing is not applicable to this article.

## Abbreviations

The following abbreviations are used in this manuscript:

ATR-FTIR	Attenuated total reflection-fourier transform infrared spectroscopy
CDT	Cytolethal distending toxin
DNA	Deoxyribonucleic acid
eDNA	Extracellular DNA
EU	European Union
EVOH	Ethylene-vinyl alcohol
HGT	Horizontal gene transfer
MP	Microplastic
Mt	Metric ton
NP	Nanoplastic
PA	Polyamide (nylon)
PAA	Polyacrylic acid
PAM	Polyacrylamide
PAN	Polyacrylonitrile
PB	Polybutylene
PBM	Poly-(n-butyl methacrylate)
PC	Polycarbonate
PE	Polyethylene
PEI	Polyetherimide
PES	Polyethersulfone
PEST	Polyester + polyethylene terephthalate
PET	Polyethylene terephthalate
PP	Polypropylene
PS	Polystyrene
PTFE	Polytetrafluoroethylene
PVC	Polyvinyl chloride
RNA	Ribonucleic acid
ROS	Reactive oxygen species
rRNA	Ribosomal RNA
SEM	Scanning electron microscopy
TW	Tap water
WWTP	Wastewater treatment plant
